# Molecular Evolution of the Bactericidal/Permeability-Increasing Protein (BPIFA1) Regulating the Innate Immune Responses in Mammals

**DOI:** 10.3390/genes14010015

**Published:** 2022-12-21

**Authors:** Hafiz Ishfaq Ahmad, Faheem Ahmed Khan, Musarrat Abbas Khan, Safdar Imran, Rana Waseem Akhtar, Nuruliarizki Shinta Pandupuspitasari, Windu Negara, Jinping Chen

**Affiliations:** 1 Department of Animal Breeding and Genetics, Faculty of Veterinary and Animal Sciences, The Islamia University of Bahawalpur, Bahawalpur 63100, Pakistan; 2 Laboratory of Molecular Biology and Genomics, Faculty of Science and Technology, University of Central Punjab, Lahore 54000, Pakistan; 3 Research Center for Animal Husbandry, National Research and Innovation Agency, South Tangerang 15314, Indonesia; 4 Laboratory of Animal Nutrition and Feed Science, Animal Science Department, Faculty of Animal and Agricultural Sciences, Universitas Diponegoro, Semarang 50275, Indonesia; 5 Guangdong Key Laboratory of Animal Conservation and Resource Utilization, Guangdong Public Laboratory of Wild Animal Conservation and Utilization, Institute of Zoology, Guangdong Academy of Sciences, Guangzhou 510260, China

**Keywords:** BPIFA1, bactericidal, immune system, molecular evolution, positive selection

## Abstract

Bactericidal/permeability-increasing protein, a primary factor of the innate immune system of mammals, participates in natural immune protection against invading bacteria. BPIFA1 actively contributes to host defense via multiple mechanisms, such as antibacterial, surfactant, airway surface liquid control, and immunomodulatory activities. However, the evolutionary history and selection forces on the BPIFA1 gene in mammals during adaptive evolution are poorly understood. This study examined the BPIFA1 gene of humans compared with that of other mammalian species to estimate the selective pressure derived by adaptive evolution. To assess whether or not positive selection occurred, we employed several different possibility tests (M1 vs. M2 and M7 vs. M8). The proportions of positively selected sites were significant, with a likelihood log value of 93.63 for the BPIFA1 protein. The Selecton server was used on the same dataset to reconfirm positive selection for specific sites by employing the Mechanistic-Empirical Combination model, thus providing additional evidence supporting the findings of positive selection. There was convincing evidence for positive selection signals in the BPIFA1 genes of mammalian species, which was more significant for selection signs and creating signals. We performed probability tests comparing various models based on dN/dS ratios to recognize specific codons under positive selection pressure. We identified positively selected sites in the LBP-BPI domain of BPIFA1 proteins in the mammalian genome, including a lipid-binding domain with a very high degree of selectivity for DPPC. BPIFA1 activates the upper airway’s innate immune system in response to numerous genetic signals in the mammalian genome. These findings highlight evolutionary advancements in immunoregulatory effects that play a significant role in the antibacterial and antiviral defenses of mammalian species.

## 1. Introduction

Bactericidal permeability-increasing protein (BPI) is a highly effective antimicrobial protein that binds and neutralizes lipopolysaccharides released from the outer membrane of bacteria [1]. The BPI fold-containing family A member 1 (BPIFA1) gene is known to have effects on the local immune system, and these effects can potentially influence the growth and invasion of microorganisms [2]. One of the potential mechanisms that underlie this link is the ability of BPIFA1 to enhance the absorption of bacteria by phagocytic cells and their ability to destroy them [3]. Although they have minimal sequence similarity, BPI has two domains that adopt the same structural fold [1,4]. Invading Gram-negative bacteria result in an integrated host response facilitated by the presence of a lipopolysaccharide-binding protein (LBP). LBP is an endotoxin-binding protein closely linked to and coordinated with BPI [5]. BPIFA1 controls the mucosal microbiota and baseline interferon signaling. SPLUNC1 (formerly known as BPIFA1) is a protein fold-containing family member with antibacterial, surfactant, and immunomodulatory activities, all of which contribute to host protection. The respiratory system is the primary site of its expression [6]. SPLUNC1, the human homolog of the mouse gene PLUNC, exhibits the same expression pattern in the upper airways and nasopharyngeal areas as its mouse homolog. Antibacterial action against Gram-negative bacteria is displayed by the encoded antimicrobial protein [5]. In non-small cell lung cancer, it might serve as a potential molecular marker for locating micrometastasis. Multiple transcript variants have been discovered as a result of the alternative splicing of the 3’ untranslated region; however, the full-length nature of only three of these transcript variants is understood [7].

Both mice and humans have significant levels of BPIFA1 gene expression in the upper part of the trachea, but this expression diminishes with distance from the trachea, reaching a minimum at the bifurcation of the main stem bronchi and becoming undetectable in the lungs’ periphery. [8]. Extensive gene expression studies in mice and humans have failed to detect BPIFA1 in peripheral lung tissue [9]. With the exception of very low levels of BPIFA1 mRNA expression in the mouse thymus, rat heart, and olfactory mucosa, BPIFA1 is not expressed in any organs or tissues outside of the respiratory system of rodents [10]. There is no indication that BPIFA1 mRNA is present in any of the following human tissues: the heart, liver, brain, stomach, small intestine, placenta, skeletal muscle, pancreas, spleen, normal lymph nodes, peripheral lymphocytes, prostate, testis, or ovary [11]. The expression of BPIFA1 mRNA follows a distribution pattern that is highly comparable in embryonic and adult tissues. This pattern is observed in both types of tissues [1]. BPIFA1 overexpression in transgenic mice produced alveolar macrophages with enhanced opsonization and phagocytosis of carbon nanotubes in a model of controlled airway inflammation [12]. In addition, commensal Gram-negative nanobacteria were shown to co-localize with BPIFA1 within the epithelial cells of nasopharyngeal cancer tissues [13]. The samples were taken from patients who had previously been identified as having nasopharyngeal carcinoma. The findings of a recent study suggested that interactions between BPIFA1 and non-bacterial LPS can mitigate the inflammatory response of the body caused by non-bacterial LPS. [1].

The range of its antibacterial effects and preservation of its structure in air-breathing vertebrates imply that BPIFA1 has evolved to provide essential host-protective capacities [14]. However, due to its location in the proximal airways and its high level under basal conditions, BPIFA1 may be indispensable. This is because antimicrobial effectors are abundant in animals [15]. Consequently, BPIFA1 seems to have the most significant effects in avoiding infection and clearing it up prior to the invasion of pathogens. Bacterial infections in the respiratory system can be prevented thanks to these functions, which may signal the activation of immunity and improved regulation of other airway functions [15].

Adaptive changes in response to environmental demands are thought to be constrained by biophysical factors, but the structural aspects of sites that contain adaptive changes cannot be predicted by any evolutionary theory [16]. This is because biophysical constraints limit the types of substitutions that are allowed for protein function to be maintained [17]. Positive selection may be more prevalent in sections of proteins where mutations are expected to have a lower effect than in other parts of the protein, although this has not been proven (e.g., allosteric regulation sites) [18]. However, functional regions are expected to remain substantially conserved during evolution, despite the fact that adaptive alterations are associated with the rapid fixation of favorable mutations [19]. The molecular evolution of protein sequences is significantly influenced by the process of natural selection. Recent developments in genome sequencing and reliable inference methods at phylogenetic and population levels have made it possible to conduct a rapid and robust assessment of the evolutionary rates and adaptations that are driven by natural selection [20]. At both the phylogenetic and population levels, a substantial amount of work has been conducted to build inference methods. Furthermore, the increasing accessibility of protein structural and functional data has allowed researchers to examine the impact of structural and functional constraints on the evolution and adaptation of protein sequences [16]. Because of the limits imposed by their structures and their functions, the rates of evolution and adaptation are different for various proteins and sites within the same protein [19].

The bulk of a cell’s functions is intricately intertwined with the regulatory networks of gene expression that enable organisms to tolerate higher infection levels or mitigate the effects of those infections [21]. Most of the components that make up cellular physiology are intimately related to these gene expression regulatory systems, which are frequently old evolutionary adaptations [22]. These mechanisms have drawn a substantial degree of interest in research that has utilized a constrained set of model species for which genetic information is available [23]. However, little is known about the mechanisms that led to the evolution of these systems or how they adapted to diverse environmental settings as evolution progressed. This study aims to investigate the evolutionary origins of the BPIFA1 gene to reveal its physiochemical features and apply comparative genomics to provide an assessment of the gene in various mammalian species. We conducted in-depth comparative studies of the bactericidal/permeability-increasing protein (BPIFA1) gene, which regulates the innate immune response in mammals, to better understand how these genes work. There is a possibility that selective pressure will have a significant effect on the evolution of adaptation. In this study, we investigate the history of these genes in various vertebrate species, as well as how genetic diversity and natural selection have influenced the development of this gene family over time.

## 2. Materials and Methods

### 2.1. Sequence Retrieval and Analysis

The amino acid and coding nucleotide sequences of the BPIFA1 gene in 34 mammalian species, including humans as the reference species in this study, were collected from GenBank (https://www.ncbi.nlm.nih.gov/genbank, accessed on 20 September 2021), and they were aligned using the Clustal Omega tool in MEGA 6 software [24]. The maximum-likelihood method was used in MEGA 6 software to generate the phylogenetic tree for the BPIFA1 gene. This tree was constructed based on the evolutionary relationships among the genes. The bootstrap test calculated the average number of substitutions per site and the average branch length by employing a maximum-likelihood method with 1000 repeats to determine taxonomic clustering. This method was used to pick a topology for more advanced log-likelihood values [25,26]. The species names and accession numbers used to study the BPIFA1 gene are provided in Supplementary Table S1.

### 2.2. Selection Analysis

Maximum likelihood approaches were used to compare the ratios of dN/dS for each codon site to identify specific codons in mammalian BPIFA1 gene sequences subjected to positive selection [27,28]. CODEML executed in PAML [29] and the DATAMONKEY webserver (https://www.datamonkey.org, accessed on 29 September 2021) [30] were utilized for the analysis, and the outcomes were designated using substitution ratios of codons that were considerably higher than 1 for codons under positive selection. The initial step of this research was to determine whether or not positive selection occurred using the maximum likelihood ratio test. This analysis determined the presence of sites with a dN/dS ratio greater than one. In this study, we contrasted a discrete (generic) model that performed this function with a null model that prohibited the occurrence of sites with a value greater than 1 [31]. Analyses were compared using a likelihood log (2Δl) distribution with df = 4. The null hypothesis (M7) asserted that the distribution was bounded by the values 0 and 1. An alternative model (M8) with two parameters, omega (ω) and beta (β), allowed for the derivation of a value from the dataset, which may be greater than 1 [27]. Analyses using fixed effect likelihood (FEL), single likelihood ancestor counting (SLAC), and random effect likelihood (REL) all found that the BPIFA1 gene was subject to positive selection when global values for synonymous and non-synonymous divergences at each site were compared [32].

The second stage was to utilize the maximum probability estimate to locate amino acid positions that were the subject of positive selection throughout the course of evolution. The Bayes theorem, which predicts the posterior probabilities of the sites that are subject to positive selection, was used to successfully accomplish this goal. Positive selection was observed to be operating at amino acid locations with posterior probabilities ranging from 95% to 99% [33]. Amino acid residues with a high probability that the value was greater than one were subjected to selective procedure. The Swiss model and Phyre 2 (http://www.sbg.bio.ic.ec.k/phyre/html, accessed on 28 September 2021) are web-based applications that display the locations of favorably selected amino acids on protein structures [34]. We predicted the location of evolutionary conservation of nucleic acids and amino acids in the protein using the ConSurf tool (http://consurftest.tau.ac.il, accessed on 28 September 2021), which was based on the phylogenetic relationship between sequences [35]. The sequence of the aligned codon of BPIFA1 was examined in Selecton version 2.2 (http://secton.tau.ac.il, accessed on 28 September 2021), which permits determining the varied ratios of various codons inside the aligned sequences. These ratios were measured using the Bayesian inference approach through various likelihood tests. This was performed to confirm positively selected codons [36]. Moreover, the Selecton results were shown in various colors to denote the various selection criteria.

### 2.3. Recombination Analysis

To find evidence of recombination, we performed a model selection procedure based on statistical likelihood that can sift through many sequence alignments in search of breakpoints and spot likely recombinant DNA. This technique used a genetic algorithm to search the alignments of several sequences for recombination breakpoints in order to accomplish its goal. The GARD approach is simple to grasp, easily extensible, and highly parallelizable. Extensive simulation experiments have demonstrated that the method beats other current tools in almost all cases, particularly concerning accuracy. To investigate the evidence of recombination, the nucleotide sequences were first assessed to identify haplotypes (*Na*) and estimate the polymorphic sites (*S*), average number of nucleotide differences (*K*), and nucleotide diversity (π) using DnaSP 5.10 software [37]. Detection of breakpoints and assessment of recombinant signals in nucleotide sequences were performed using the online GARD tool of the Datamonkey webserver [38]. Additionally, using GARD to screen sequences for recombination assures that methods focused on identifying positive selection have acceptable statistical features.

### 2.4. Protein-Protein Interactions Analysis

Much interest has been directed toward investigating how protein-protein interactions are preserved from one species to another. Since there are several hurdles in the experimental identification and confirmation of interactome data, it would be intriguing to understand a PPI transferred from a species that has been proven in another species [39]. The STRING databank is a free bioinformatics resource that contains information describing how proteins interact with one another as part of several pathways. The number of lines connecting each protein node and betweenness values are used to identify intermediate nodes, representing proteins that play important biological roles and are intimately linked to one another. Network creation was carried out using STRING and Cytoscape software (http://www.cytoscape.org, accessed on 29 September 2021) was used to display the network [40]. By identifying the protein-protein interactions of BPIFA1 among immune proteins and co-expression analysis using STRING version 9.1 (http://www.string-db.org, accessed on 29 September 2021), we were able to further determine how BPIFA1 functions at the molecular level.

### 2.5. Structural Analysis of BPIFA1 Protein

In this analysis, we built the crystal structure of the human BPIFA1 protein using homology modeling with online tools, such as the Swiss model (http://swissmodel.expasy.org, accessed on 29 September 2021) [41], I-TESSAR [42], and Phyre2 (http://www.sbg.bio1.ic.ac.uk/phyre2/html, accessed on 29 September 2021) [43]. The conjugate gradient method and Amber force field in UCSF Chimera 1.10.1 software were used to reduce the assembled target protein. In addition, the ProSA webserver was utilized to evaluate the stereochemical properties of the expected structure [22].

## 3. Results

The BPIFA1 protein sequences encoded in the mammalian genome were studied to determine the role of adaptive selection and evolution. The protein BPIFA1 is the key mediator of innate signaling against microbial infections by bacteria and fungi. Once the sequences were combined using MSA, they were utilized to create Bayesian phylogenetic trees and undergo further investigation. To initiate intracellular signaling cascades, activating a set of genes identified in the appropriate mammalian species and possessing a functioning (LBP-BPI) domain is necessary. For the surfactant phospholipid dipalmitoylphosphatidylcholine (DPPC), this lipid-binding domain has a very high degree of selectivity. The upper airway’s innate immune system is activated in response to numerous genetic signals, such as increased non-synonymous substitution rates, significant homologous haplotypes, and an absence of genetic variation in BPIFA1 proteins, demonstrating that the presence of these proteins has been favored by positive selection.

### 3.1. Molecular Evolution of BPIFA1 Gene

**Figure 1:**
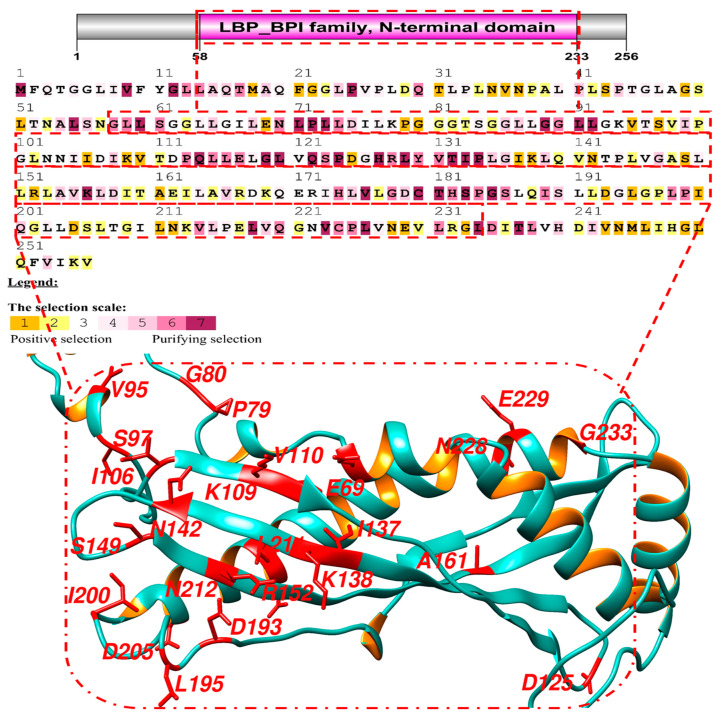
Results of adaptive selection on 20 primate BPIFA1 sequences using the MEC model. The human protein was used as a reference. Positive selection is indicated by yellow and magenta, whereas purifying selection is represented by blue and green.

In this work, we searched for signs of adaptation in the BPIFA1 gene, ranging from progressively weak to strong selection signals during adaptive evolution in the mammalian genome. The typical percentage of codons in the BPIFA1 gene undergoing adaptive evolution was determined. Following the same procedure for each coding sequence, we calculated the average proportion of positively selected codons across all branches. Using BUSTED and synonymous rate variation in carefully chosen test branches of the BPIFA1 phylogeny, we determined traces of gene-wide episodic diversifying selection. As a result, we concluded that divergent selection occurred along the three examined lines of descent. Using synonymous rate variation, we observed gene-wide episodic diversifying selection in the test branches of the BPIFA1 phylogeny. A gene-wide episodic diversifying selection was used to achieve this (LRT). Two test branches exhibited evidence of diversifying selection, suggesting that the site had been subjected to this type of evolution (Figure 1).

The average dN/dS ratios for BPIFA1 across all sites and lineages were greater than one. As a result, research was conducted on this protein to identify the signatures of positive selection. The protein was found to have a conserved structure of amino acids, making it possible to be purified, and it had an omega value greater than 1. A log-likelihood test was performed on this protein, all of its sites were analyzed, and the substitution rate was calculated. To assess whether or not a positive selection occurred, we used three different sets of likelihood models: M0 vs. M3, M1 vs. M2, and M7 vs. M8. The parameter estimates under M1 and M2 were compared and it was found that the M2 value for these proteins was positive. The percentages of positively selected sites were significant for the three models, with values of 422.86, 64.5, and 93.63, respectively (Table 1). To provide additional evidence to support the findings of positive selection, we applied the Mechanistic-Empirical Combination model to specific sites using the Selecton server. During this process, we discovered that several sites had been identified as having been subjected to selective pressure at various points during evolution (Figure 1). Because of this, we could estimate the degree to which this gene has been evolutionary conserved. We found that the vast majority of the positively selected sites had been conserved throughout the mammalian clades. This was because the conserved amino acids accounted for most of the signals used for positive selection in the neural network’s algorithm (Table 2).

**Figure 2:**
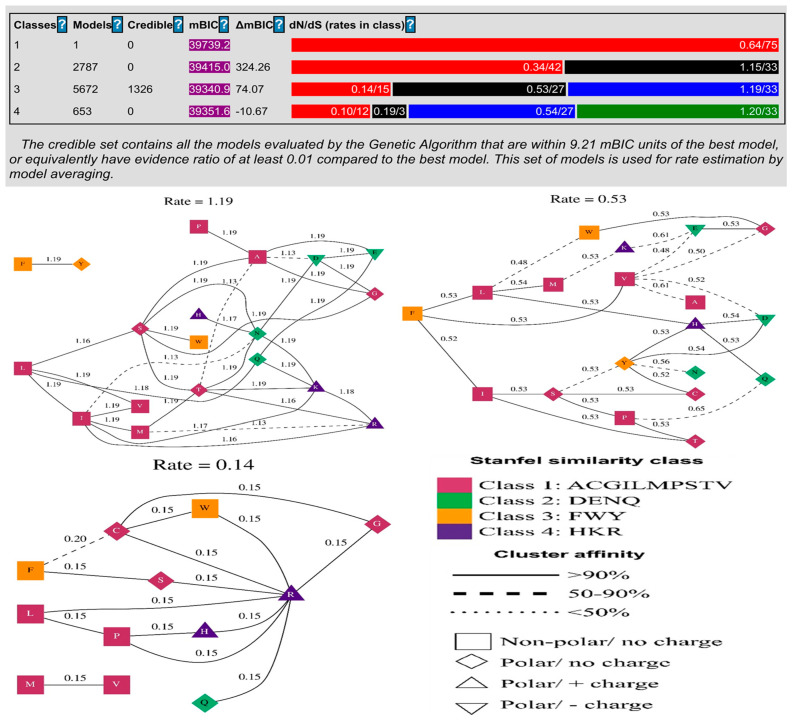
Application of a genetic algorithm (GA) model to identify structural and evolutionary rate clusters from BPIFA1 protein alignments. Maximum-likelihood estimation was used to identify each cluster and GA was used to determine its rate.

The codon model selection method evaluated 9113 different models. The best model (log(L) = −18,910, mBIC = 39,340.92) contained three rates and was the most accurate. With this model, improvements of 218.66 log(L) and 398.33 mBIC points were achieved compared to a single rate model, in which all non-synonymous substitutions occurred at the same rate, as shown in Table 1. Each model in the credible set had an evidence ratio of at least 0.01 compared to the best model, meaning that it was within 9.21 mBIC units of the best model, or equivalently, that it had an evidence ratio of at least 0.01 compared to the best model. Model averaging estimated the rate of change in this collection of models (Figure 2). The evolutionary selection pattern on amino acid positions in the BPIFA1 protein was also assessed using codon model selection analysis, which showed that the substitution of amino acid sites occurred during adaptive evolution in the proteins. We revealed that the basic amino acid positions of the proteins exhibited adaptive evolution due to varying substitution rations. Based on the distribution of amino acid sites in BPIFA1, the maximum substitution rate was approximately 1.19, while the lowest was.14 (Figure 2).

Identification of physiologically significant regions of a protein can be performed by contrasting the frequency of synonymous (Ks) and non-synonymous (Ka) substitutions in the protein. This provides the basis for concluding the existence of purifying selection and localized positive Darwinian selection. We used Selecton v. 2.2 (accessible at http://selecton-bioinfo-tau.ac.il, accessed on 29 September 2021), a web server that automatically calculates the ratio of Ka to Ks (u) at each site in the protein. Different colors represent different types of selection (positive selection, purifying selection, and no selection) and are used to graphically display this ratio at each site. The Selecton model is a collection of different evolutionary hypotheses that can be used to statistically test the likelihood that a given protein has been subjected to positive selection. It operates via a graphical user interface. The recently established mechanistic-empirical model influenced the amino acid’s physical properties (Table 3).

### 3.2. Adaptive Selection of BPIFA1 Gene

**Figure 3:**
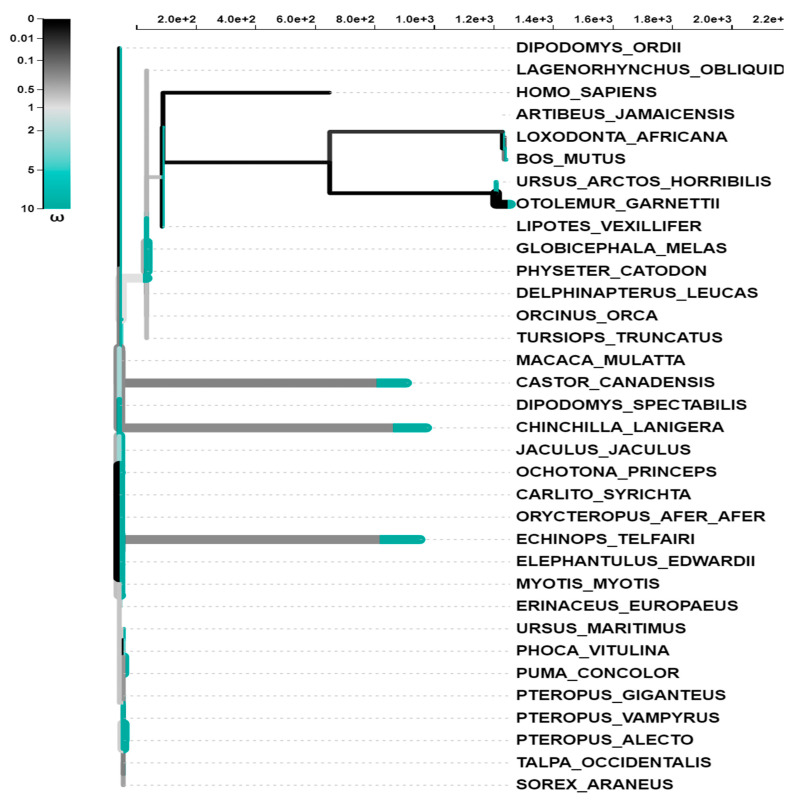
The aBS-REL models used to undertake selective analyses of vertebrate activating transcription factor genes. The length of the branch is separated into segments based on the percentage of sites that correspond to each class, and the color of the branch segment shows the relative relevance of the relevant parameters. Because of this, sites along a branch can be categorized according to the β distribution that has been inferred. Depending on whether or not the site has a *p* value of less than 0.05 (adjusted for multiple testing), thicker branches are categorized as having either undergone diversifying positive selection or diversifying negative selection. This determination is based on whether or not the null hypothesis is rejected.

To determine the degree to which different mammalian species have adapted to their environments, we used multiple alignments of the coding sequences of the BPIFA1 gene from each of the 34 species. These tests can be employed individually or in combination. The most common variety of tests is known as a branch test. During evolution of the vertebrate species, the selection of specific lineages was utilized to recognize distinct lineages as subject to selection pressure. Lineage-specific selection probabilities were calculated for each phylogenetic group using an adaptive branch-site random effects likelihood (aBS-REL) model. In addition, the aBS-REL technique was utilized to dissect each gene to determine which lineages had been subjected to adaptive selection at different times in evolutionary history. When applied to mammalian lineages, the aBS-REL model confirmed that the BUSTED-predicted genes were under positive selection. Our results, which suggested that selective pressure was acting on BPIFA1 genes in mammalian lineages, demonstrated that the two hypotheses were congruent (Table 4). In the phylogeny of the BPIFA1 gene, there was evidence of episodic diversifying selection in eight branches. The importance of the findings was evaluated using the Likelihood Ratio Test (*p* > 0.05), which was carried out after the outcomes of many other tests were considered (Figure 3). In total, 63 distinct lines were put through this specific test for diversifying selection. Multiple tests were carried out, and the significance of the findings was established by applying the Likelihood Ratio Test with a *p*-value threshold of 0.05.

This table reports a statistical summary of the models’ fit to the data. Baseline MG94xREV refers to the MG94xREV baseline model that infers a single ω rate category per branch. The full adaptive model refers to the adaptive aBS-REL model, which implies an optimized number of ω rate categories per branch.

During the evolutionary process, we examined the omega values by employing the SLAC, FUBAR, MEME, and FEL methods to locate indications of positive selection (Table 5). According to our findings, the BPIFA1 gene in mammalian clades has been subject to positive evolutionary selection. We could detect which regions of the genome were being subjected to selective pressure by using the Bayesian method. This technique involves determining the posterior probability for each codon. Sites with a greater number of possibilities are more likely to have undergone diversifying selection, which leads to higher rates of non-synonymous and synonymous substitution than sites with a lower number of probabilities (Table 2). Using BEB analysis, we found that several locations all across the bactericidal protein’s LBP-BPI domain had been subject to positive selection with a high posterior probability of 95%. This was the case for all sites. The sites were dispersed throughout the domain in various locations. The findings of PAML were examined using the dataset found in the Selecton server. This server was able to identify adaptive selection at certain sites within the protein, which allowed us to validate the existence of positive selection. To determine the substitution rates, the MEC model was applied. The findings demonstrated that adaptive selection occurred at several locations in BPIFA1 (Table 5).

### 3.3. Recombination Analysis

**Figure 4:**
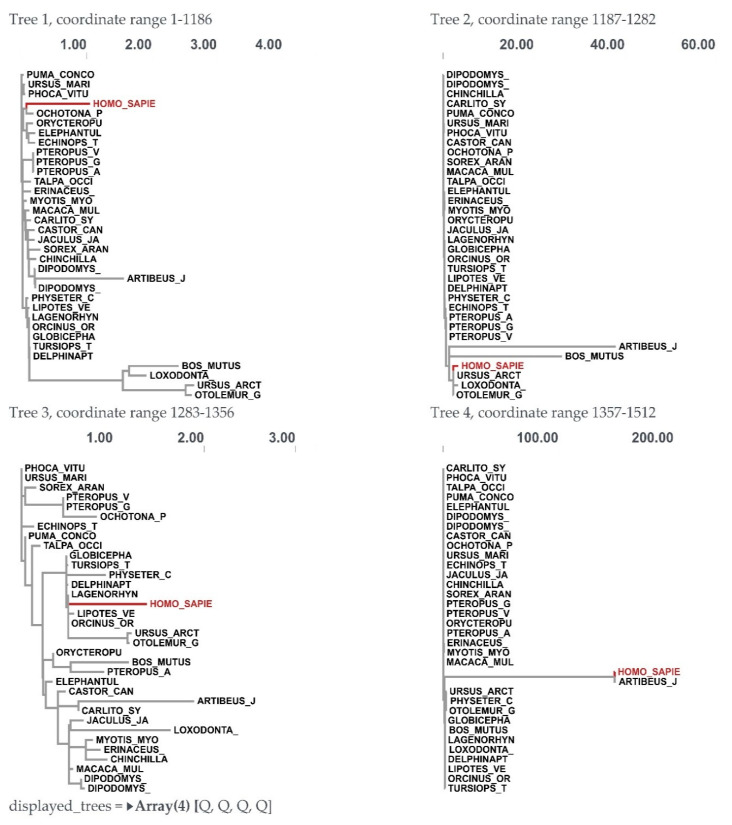
Trees for individual fragments showing recombination breakpoints in the BPIFA1 gene among mammalian species.

**Figure 5:**
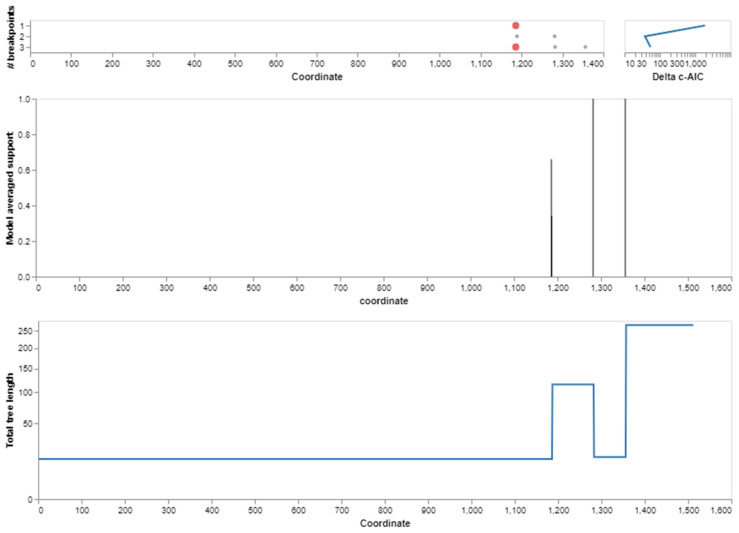
Breakpoints detected in the BPIFA1 gene during evolution. The location of breakpoints, as determined by the algorithm, for each of the different numbers of breakpoints that were taken into consideration. The progression of the c-AIC score from one set of breakpoint numbers to the next (log scale).

For the BPIFA1 gene, a recombination analysis was performed to find potential evolutionary links between genes. The research revealed three recombination events. Each of the recombination sequences, including the major and minor parents, came from the BPIFA1 gene. We identified recombination breakpoints using GARD analysis. At a rate of 30.30 models per second, GARD inspected 5120 models. The search space of 72,874,879 models with up to three breakpoints was generated by the alignment’s 759 possible breakpoints, of which the genetic algorithm only examined 0.01%. With an evidence ratio of 100 or above, the multiple tree model was preferred to the single tree model, indicating that at least one of the breakpoints actually reflected a topological incongruity. This was validated by comparing the AICc scores of the best-fitting GARD model, which allowed for variable topologies across segments (37,996.2), and the model, which assumed the same tree for all of the partitions determined by GARD, but allowed varied branch lengths between partitions. Specifically, the AICc score of the best-fitting GARD model was 37,996.2, whereas the AICc score of the model was 37,996.2. (Figure 4 and Figure 5).

### 3.4. Protein-Protein Interactions and Ligand Binding Analysis

**Figure 6:**
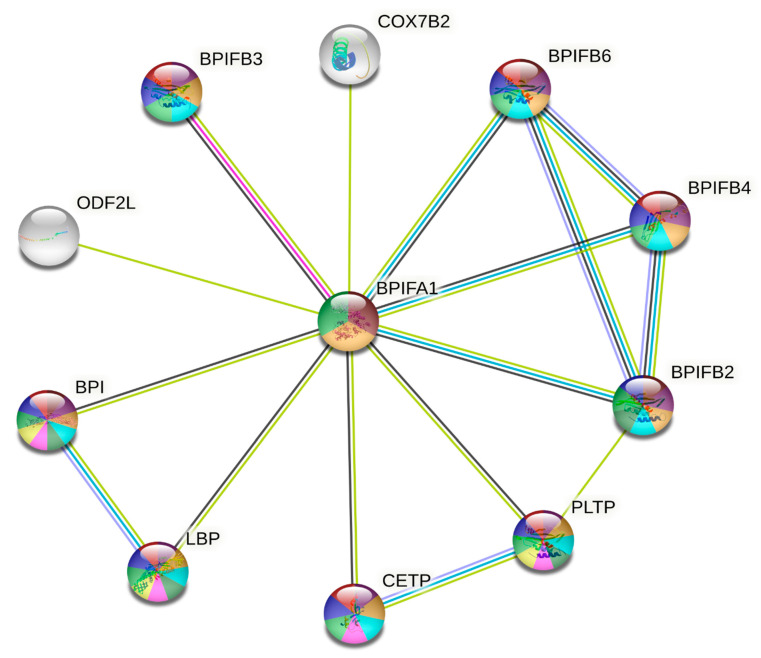
The protein-protein interaction (PPI) network for the BPIFA1 gene constructed using the online STRING database. The genes that are responsible for upregulation, downregulation, and neutral regulation are represented by red, blue, and green circles, respectively. The intensity of the interactions that take place between these genes is represented by the thickness of the lines that connect them. Mean values of a negative correlation coefficient are represented by solid edges, whereas mean values of a positive correlation coefficient are represented by dotted lines. Changes in the folding or stitching of proteins that take place after transcription are represented as nodes in the protein-protein interaction (PPI) network. Each node in the network represents the whole set of proteins that can be produced by a single copy of the protein-coding gene.

We used the STRING database to search for proteins expressed with BPIFA1, identifying several pairs of protein-protein interactions. There were 13 nodes and 35 edges denoted by the proteins expressed with BPIFA1. The edges of the PPI diagram are the line networks that link the individual nodes (Figure 6). The average local clustering coefficient value was 0.978. PPI enrichment had a *p*-value of 5.25 × 10^−12^. The PPI network represented the BPIFA1 gene’s interactions with other co-expressed immune genes. COX7B2, BPIFB6, BPIFB4, BPIFB2, BPIFB3, PLTP, CETP, BPI, LBP, and ODF2L were the 10 genes involved in the PPI network of BPIFA1 (Figure 6).

The BPIFB6, BPIFB4, BPIFB2, and BPIFB3 genes were the most significant because they are involved in biological signaling pathways, which play an essential role in innate immunity against bacterial infection. In addition, these genes are upregulated by BPIFA1, which is another reason they were considered so significant (Table 6). The molecular pathways essential in eradicating invading germs through membrane-disrupting activity comprised all related proteins with varied roles. Membrane-disrupting activity was necessary for the elimination of invading germs. Two crucial proteins in the mediation of signals in response to lipopolysaccharides include LPS-binding protein (LPSBP) and bactericidal permeability-increasing protein (BPI). They displayed a strong affinity for Lipid A, a substance found in LPS, and were strikingly similar to one another. Despite having similar structures, LBP and BPI perform various biological functions that are distinctly different from one another. For instance, LBP frequently binds to LPS and greatly facilitates the presentation of LPS to CD14+ cells, such as macrophages and monocytes, whereas BPI inhibits and lowers the bioactivity of LPS. These two proteins are both present in bacteria.

**Figure 7:**
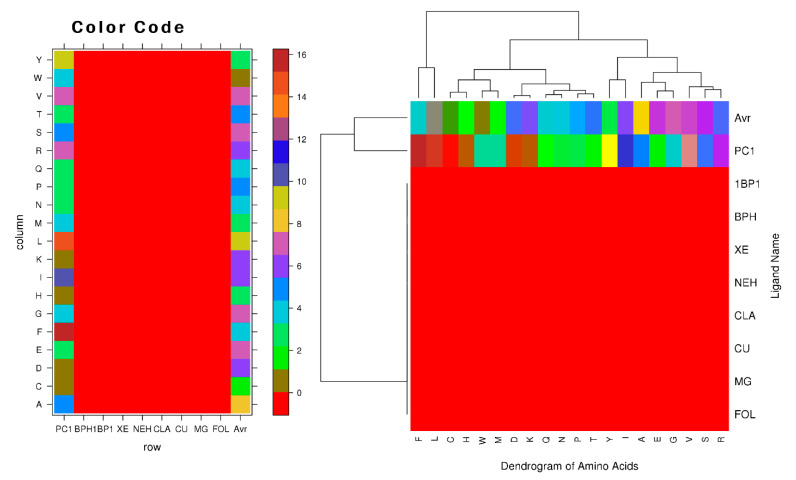
Clustering of amino acids according to the physical features of ligand-interacting amino acids (**right**) and the residue composition of various ligand binding sites (**left**).

Ligands are critical components in the process of controlling the expression and activity of proteins. Intermolecular binding forces, such as ionic bonds, hydrogen bonds, hydrophobic interaction, and Vander-Waals forces, contribute to the ligand-binding process. Due to interactions between ligands and proteins, the protein’s three-dimensional structure will be altered. Because of these changes in the conformational state of the protein, some of the protein’s functions may be either inhibited or activated. Therefore, we performed a protein-ligand binding interaction study using amino acid physiochemical characteristics to determine which residues interact with the ligand and which do not. To accomplish this, we used a website (http://crdd.osdd.net/raghava/lpicom, accessed on 18 October 2021) that calculates the fraction of residues that interact with a given ligand. Key residues, such as cysteine, glycine, alanine, lysine, aspartic acid, histidine, leucine, valine arginine, tryptophan, serine, threonine, and tyrosine, were shown to interact with seven ligands (1BP1, BPH, XE, NEH, CLA, CU, and MG) and PC1. Compared to the interaction with PC1, charged amino acids, especially essential amino acids, had a greater advantage when interacting with 1BP1, BPH, XE, NEH, CLA, CU, and MG (Figure 7). The small and polar amino acids that correlated with them were characterized in each of the three ligands. We used two distinct approaches to make predictions regarding complementary binding sites: the first was predicated on comparing binding-specific substructures (TM-SITE), while the second was predicated on the alignment of the sequence profiles (S-SITE). These techniques assessed the BPIFA1 protein against 500 non-redundant proteins that combined with 814 organic, synthetic, and metal ion compounds. Beginning with predictions of low-resolution protein structures, the approaches successfully identified the binding residues of BPIFA1, achieving an average Matthews correlation coefficient (MCC) that was much higher. Additionally, the techniques uncovered ligands that bind with the residues (Table 7).

## 4. Discussion

Heterogeneous backgrounds offer platforms where populations undergoing divergent selection can be distinguished into natively adapted subpopulations [44]. The influence of selection on gene flow among populations, such as migration-selection balance, determines the possibility of innate adaptation and continued divergence. This is also known as the migration-selection balance. There is a tendency for local genetic variability within populations to become homogenized due to gene flow when the effect of selection is less significant than the effect of gene flow. Instead, genetic variants may accumulate and be retained across specific loci susceptible to powerful divergent selection if the selective pressure is greater than the integrative force of gene flow [45]. In the possible alternative outcome, the benefits of gene flow are limited by selection against immigrants who have a poor genetic fit, which also paves the way for local adaptation [45,46]. There must be a connection between gene flow and selection to understand population differences in the frequency of gene flow [46]. Under such circumstances, selection determines whether the population continues to evolve or diverge as a distinct group. The empirical Bayes approach calculated the LRT at each branch site and located all the different sites where diversified selection may occur. Based on the empirical Bayes approach, the Fast, Unconstrained Bayesian Approximation, also known as FUBAR, was applied to locate the diversifying selection occurring at the BPIFA1 gene. FUBAR allowed for site-to-site and branch-to-branch dispersion of codons and was utilized to explore the adaptive evolution that occurred at the gene level. The method of MEME was utilized to investigate the adaptive evolution that occurred at the gene level [25,32,47]. The episodic diversifying coding sites were found by SLAC with a *p* value of less than 0.01 (Table 1). This model was used to estimate the synonymous and non-synonymous substitution rates, and coding sites with synonymous substitution rates greater than or equal to the non-synonymous rate were considered noteworthy for identifying sites that were undergoing diversifying selection. In MEME, maximum-likelihood estimations for the BPIFA1 gene’s codons 130, 167, 168, 190, 243, 265, and 289 were obtained (Table 2). Based on their non-significant signals, these codons were not identified as positively selected sites, which is due to the episodic character of natural selection. The natural selection that took place sporadically throughout brief intervals of adaptive evolution was masked by the frequent occurrence of either purifying or natural selection. Consequently, signs of adaptive evolution could not be found via sensitivity testing and positive selection [48].

We found seventeen sites that were favorably chosen using the PAML method, fifteen sites that were chosen using the IFEL algorithm, and four sites that were chosen using the FEL algorithm. The adaptive selection pressure on the BPIFA1 gene’s codon sequences was calculated using the MEC model. This resulted in the identification of seventy-four amino acids (Figure 1). A model of evolution based on positive selection was used, revealing differences at the codon level (M8). The MrBayes application on the Selecton server utilized an MCMC model to previously determine differences in the MAVS gene in mammals at the codon level [49]. Based on the results of MAFFT protein alignments, previous studies have shown that the Ig domain remains in the MAVS coding sequences. These results suggest that alternative protein switches in purifying selected regions are deleterious and thus unlikely to be maintained throughout evolution [50,51]. Sites for multiple evolutionary pathways were identified using a multi-parameter rate distribution, a random effect model with a 95% confidence interval, and substantial Pr [β > α] values. Sites could then be located thanks to this method (Table 3). In the case of positive selection, the class rate weight was determined using a bivariate general discrete distribution for each coding site. Convergence of the MCMC model was demonstrated by the fact that the posterior mean estimates for BPIFA1 were found to be closer to the considering reduction factor value (Table 2). These values ranged from 0.95 to 0.99. During the process of diversifying selection, only the coding sites with empirical Bayes factor (EBF) values of more than 50 were considered. Calculations were performed using the net effective sample size to determine the EBF values for each coding site evaluated using positive selection. Inferring the distribution of gene-specific selection parameters could improve the detected selections across a large number of coding sites. The coding areas that were positively selected and identified give significant evidence of diversifying selection in BPIFA1 genes that are now undergoing selective lineage. As a result, some mutations that initially appear to be neutral (and have no immediate impact on fitness) can be “permissive,” allowing the protein to withstand later changes that would otherwise be harmful and cause phenotypic differences [52]. Neutral mutations in epistasis lay the foundation for later selection and adaptation, which has recently attracted much attention and been offered as a way to reconcile neutral and selection models of evolution [53].

The substitution rate for the pair FWY and HKR was approximately 50%, the substitution rate for DENQ was 50%, and the substitution rate for ACGILMPSTV was 90%. The PPI network represented the interactions of the BPIFA1 protein with other co-expressed immune proteins. COX7B2, BPIFB6, BPIFB4, BPIFB2, BPIFB3, PLTP, CETP, BPI, LBP, and ODF2L were the ten genes that we determined to be responsible for these protein interactions (Figure 6). The BPIFB6, BPIFB4, BPIFB2, and BPIFB3 genes are the most significant because they are involved in biological signaling pathways, which play an essential role in innate immunity against bacterial infection. In addition, these genes are upregulated by BPIFA1, providing another reason that they are so significant (Table 6). Interfaces contain clusters of conserved residues with an amino acid composition compatible with both the interface core (residues with the largest change in burial upon binding) and a conserved region [54], and hot regions evolving from the clustering of hot spots correspond to tightly packed and conserved regions. Thus, interfaces are under evolutionary pressure to sustain current connections while averting unfavorable, non-specific interactions. Certain physicochemical features can be altered to reduce the likelihood that protein-protein interfaces may form dysfunctional interactions [55]. As a result of our investigation, we found that values were more than 1 for positively selected codons presented in Table 1. This illustrates that the development of synonymous sites required more time than the development of non-synonymous sites (dN sites). This beneficial impact of Darwinian selection, which encourages novel variations and greater allelic polymorphism, operates as balancing or purifying selection [56], which causes an alteration in the structural protein and affects the signaling pathway [57]. In spite of the fact that they originate from the same lineage, amino acid substitutions in the offspring of different species might have very different consequences [56,57]. This contrasts with the fact that their pedigree coincides with earlier submissions. The BPIFA1 genes chosen in this study provide some information for bioanalysis, which aims to select genes based on the evolutionary time scale from the most recent to longer-term periods. In addition, the fundamental evolutionary mechanism that has been uncovered as a result of recent research may be insufficient due to the absence of the structural and functional features of a large number of proteins in the genome. The evolution and adaptation of protein-coding genes in *Drosophila melanogaster* were thoroughly examined in order to determine the most relevant determinants of evolution and adaptation at the level of protein-coding genes. This was accomplished by comparing *D. melanogaster* to closely related species and their own populations. Large-scale applications of bioinformatics and structural analysis were carried out by our team in order to ascertain the structural and functional features of proteins. Subsequently, we divided the residues into a variety of structural and functional sites using our categorization system. The rates of sequence evolution and adaptation were compared across a variety of proteins and locations, which enabled the identification of hotspots of adaptation across the whole genome. In addition, it has been demonstrated that fast-adaptive proteins interact with one another at rates that are higher than what would be predicted by chance; this discovery shows that coadaptation is likely ubiquitous among fast-adaptive proteins.

As a result of their physical connections, the following are examples of mechanisms that have the potential to contribute to coadaptation: (1) fast-adaptive proteins are often found to be enriched in similar chemical activities and exposed to similar selection pressure, and (2) fast-adaptive proteins coevolve. Two different instances of adaptive evolution in PPIs were demonstrated in this research, which leads the authors to hypothesize that these physical interactions may have played a role in the coadaptation of fast-adaptive proteins in *D. melanogaster*. In addition, we showed that the phenomenon of coadaptation may take place in a more general sense than only between fast-adaptive proteins. The rate of adaptation is typically higher in proteins that interact with fast-adaptive proteins. Given that molecular interactions play a role in adaptive evolution, it is fair to anticipate that these interactions may also govern coadaptation at a more global level. It has been postulated that the coevolution of physical contacts is the mechanism responsible for the similar evolutionary rates observed in interacting proteins.

## 5. Conclusions

Our goal was to identify the selective pressures that have contributed to the development of the plant and mammalian BPIFA1 system, the expression of which is modulated in a wide variety of diseases. The BPIFA1 protein rapidly evolved in response to selective pressure in the human lineage, and we were able to pinpoint the genetic selection determinants that account for its bactericidal activity. During its evolutionary history, positive selection may have had a crucial role in improving the virulence response to different stimuli, which could explain the observed diversity in the stability of the gene’s function. Our findings provide a more comprehensive understanding of the evolutionary history of BPIFA1 genes, which will enhance the functional genomics analysis of pathogenicity in biological processes. It is anticipated that these findings may also help to improve the understanding of disease prevention. Additionally, the study of these genes might facilitate the design of a unique method that could assist in determining the various virulence proteins present in bacterial pathogens. Our findings lead us to hypothesize that restrictions during the evolutionary process have played a key role in shaping our discoveries. As a result of these limitations, we were able to identify some numerical boundaries when we coupled characteristics such as protein length to complicated complexes. The unique characteristics of proteins are intriguing because they may provide an indication of unusual stressors or homeostatic adjustments that have enabled their presence in cells. Therefore, they are a promising choice for further research.

## Figures and Tables

**Table 1:** Log-likelihood tests and statistics for positive selection among codons in CODONML in PAML using the codon frequency model: F3x4.

Gene	n	Lc	S	Model	*lnL*	2Δl M3 vs. M0	2Δl M2 vs. M1	2Δl M8 vs. M7	Parameter Estimates	PAML Site Model (M8) ω > 1	SLAC (*p* < 0.05)	FEL (*p* < 0.05)	FUBAR (post pr. 0.9)
BPIFA1	34	504	49	M0 (one-ratio)	−18,981.403	422.86	64.5	93.63	one ratio	125, 129, 130, 178, 184, 242, 259, 270, 304, 385, 401, 402, 425, 434, 437	130, 270, 385, 402, 425, 455	130, 259, 402, 425	130, 270, 385, 402, 425
				M1 Nearly Neutral (2 categories)	−18,802.441				p1 = 0.45328, p2 = 0.54672				
									ω 1 = 0.24322, ω 2 = 1.00000				
				M2 Positive Selection (3 categories)	−18,770.141				p1 = 0.43064, p2 = 0.45653, p3 = 0.11283				
									ω 1 = 0.26160, ω 2 = 1.00000, ω 3 = 2.62492				
				M3 Discrete (3 categories)	−18,769.971				p1 = 0.41335, p2 =0.46115, p3 =0.12550				
				M7 β (10 categories)	−18,829.824				p = 1.04797, q = 0.59014				
				M8 β&Ꙍ > 1 (11 categories)	−18,783.01				p0 = 0.78568, p = 1.86405 q = 1.74778				
									p1 = 0.21432, ω = 1.93377				

**Table 2:** Positively selected locations under the PAML model are discovered using Bayes empirical Bayes (BEB) analysis.

Gene	Model	Positively Selected Sites	Amino Acids	Posterior pr. (ω > 1)	Post Mean ± SE for w
BPIFA1	M8 β&Ꙍ > 1 (11 categories)	71	P	0.555	1.652 ± 0.876
		94	H	0.503	1.548 ± 0.897
		106	H	0.618	1.762 ± 0.863
		125	G	0.962 *	2.356 ± 0.403
		128	Q	0.938	2.318 ± 0.463
		129	D	0.998 **	2.414 ± 0.283
		130	P	0.615	1.787 ± 0.793
		172	L	0.874	2.214 ± 0.580
		177	W	0.907	2.268 ± 0.529
		178	E	0.963 *	2.359 ± 0.399
		184	A	0.997 **	2.412 ± 0.288
		190	G	0.764	2.030 ± 0.721
		192	L	0.634	1.795 ± 0.842
		207	V	0.795	2.084 ± 0.686
		208	S	0.812	2.113 ± 0.661
		213	L	0.652	1.829 ± 0.833
		216	H	0.669	1.864 ± 0.809
		242	Q	0.989 *	2.399 ± 0.320
		260	G	0.621	1.799 ± 0.788
		262	V	0.992 **	2.404 ± 0.309
		270	N	0.999 **	2.416 ± 0.278
		304	L	0.975 *	2.377 ± 0.367
		308	E	0.633	1.816 ± 0.789
		356	P	0.908	2.270 ± 0.527
		385	Q	0.980 *	2.385 ± 0.349
		390	V	0.531	1.650 ± 0.798
		401	H	1.000 **	2.416 ± 0.277
		402	Q	0.991 **	2.403 ± 0.313
		403	L	0.68	1.891 ± 0.777
		417	S	0.937	2.316 ± 0.463
		432	E	1.000 **	2.416 ± 0.278
		434	Q	0.993 **	2.406 ± 0.304
		436	W	0.964 *	2.360 ± 0.395
		437	G	0.969 *	2.368 ± 0.383
		452	L	0.808	2.104 ± 0.671
		459	C	0.702	1.921 ± 0.788
		460	A	0.511	1.584 ± 0.864
		468	T	0.697	1.903 ± 0.814
		469	Q	0.705	1.925 ± 0.794

(*: *p* > 95%; **: *p* > 99%).

**Table 3:** Sites under episodic diversifying selection inferred by MEME.

Codon	α	β-	Pr. [β = β−]	β+	Pr. [β = β+]	*p*-Value	*q*-Value
130	0.0000	0.0000	0.2042	3.4440	0.7957	0.0078	0.2638
167	0.4663	0.1038	0.7853	5.3438	0.2146	0.0066	0.2596
168	0.7252	0.2013	0.8497	28.492	0.1502	0.0008	0.0612
190	0.0000	0.0000	0.7071	14.030	0.2928	0.0035	0.1774
243	0.3055	0.0426	0.8016	3.2988	0.1983	0.0086	0.2561
265	0.6382	0.0000	0.7209	9.2337	0.2790	0.0086	0.2720
289	0.3685	0.0771	0.9177	15.304	0.0822	0.0052	0.2184
313	0.2462	0.2462	0.8605	21.499	0.1394	0.0071	0.2576
314	0.4211	0.3321	0.8708	70.669	0.1291	0.0001	0.0160
391	1.0888	0.3282	0.9122	105.28	0.0877	0.0041	0.0070
401	1.4302	0.8308	0.8019	364.04	0.1980	0.0096	0.2549
402	0.3620	0.3125	0.3515	25.937	0.6484	0.0040	0.1878
403	0.4970	0.4970	0.9236	442.56	0.0763	0.0021	0.1190
425	0.0000	0.0000	1.0000	1.1829	1.0000	0.0020	0.1319
432	0.8816	0.3808	0.7310	46.308	0.2680	0.0005	0.0502
437	0.9684	0.0118	0.7491	24.911	0.2508	0.0031	0.0090
452	0.3900	0.3654	0.8800	330.91	0.1199	0.0001	0.0145
455	0.4795	0.2852	0.8823	1362.1	0.1176	0.0051	0.0044
457	0.5413	0.0000	0.7556	10.723	0.2443	0.0088	0.2471

The distribution of synonymous (α) and non-synonymous (β) substitution rates across sites estimated by the MEME model is shown in this summary table, where the percentage of branches with β > α is much higher than 0. The *p*-value was calculated using a combination of χ^2^ distributions. The Simes technique regulated the false discovery rate under the strict neutral null and generated the q-values (likely to be conservative).

**Table 4:** Sites under episodic diversifying selection inferred adaptive Branch Site REL (aBS-REL model).

Name	B	LRT	Test *p*-Value	Uncorrected *p*-Value	*ω* Distribution over Sites
Node43	0	30.9022	0.000	0.000	ω1 = 1.00 (90%)
					ω2 = 568 (9.7%)
Node48	0	57.7808	0.000	0.000	ω1 = 0.632 (90%)
					ω2 = 1000 (10%)
Node58	0	35.478	0.000	0.000	ω1 = 0.784 (82%)
					ω2 = 38.9 (18%)
OTOLEMUR_GARNETTII	0	35.1949	0.000	0.000	ω1 = 0.00 (84%)
					ω2 = 3850 (16%)
PHYSETER_CATODON	0	46.7818	0.000	0.000	ω1 = 0.498 (85%)
					ω2 = 37.2 (15%)
PTEROPUS_ALECTO	0	39.4366	0.000	0.000	ω1 = 1.00 (96%)
					ω2 = 881 (4.3%)
Node9	0	22.9271	0.0002	0.000	ω1 = 1.00 (85%)
					ω2 = 9090 (15%)
ECHINOPS_TELFAIRI	0	22.2294	0.0003	0.000	ω1 = 0.262 (87%)
					ω2 = 1000 (13%)
PUMA_CONCOLOR	0	20.5045	0.0006	0.000	ω1 = 1.00 (98%)
					ω2 = 220 (2.2%)
OCHOTONA_PRINCEPS	0	16.9285	0.0038	0.0001	ω1 = 0.486 (85%)
					ω2 = 102 (15%)
Node1	0	15.9753	0.0061	0.0001	ω1 = 0.00 (96%)
					ω2 = 543 (3.5%)
Node42	0	15.9466	0.0061	0.0001	ω1 = 0.00 (97%)
					ω2 = 1000 (3.0%)
CHINCHILLA_LANIGERA	0	15.7558	0.0065	0.0001	ω1 = 0.260 (89%)
					ω2 = 1000 (11%)
CASTOR_CANADENSIS	0	14.0154	0.0154	0.0003	ω1 = 0.217 (90%)
					ω2 = 1000 (10%)

**Table 5:** Positive selection sites using IFEL.

Codon	dS	dN	dN Leaves	dN/dS	Normalized dN-dS	*p*-Value
85	9.97170	4.39904	0.85722	4.4116	0.3100	0.090973
105	0.22288	19.2224	0.00000	86.244	1.33923	0.050153
113	0.16321	1.21552	1.14983	7.4470	0.07417	0.076543
130	0.02672	1.38927	3.15186	51.992	0.09604	0.050535
168	0.97226	3.28401	0.62962	3.3780	0.16295	0.086927
172	0.87119	3.38718	1.12971	3.8880	0.17734	0.069473
207	0.71507	4.49186	0.73823	6.2820	0.26621	0.007236
271	0.45387	9.03290	0.52358	19.902	0.60471	0.037150
346	0.00000	1.01879	0.08543	Infinite	0.07181	0.027248
385	0.36337	3.73078	1.22177	10.267	0.23736	0.017551
390	0.52418	2.17195	1.06171	4.1430	0.11614	0.054228
402	0.53356	38.1391	1.54115	71.480	2.65073	0.037807
410	0.30457	2.07557	0.82994	6.8150	0.12483	0.052322
425	0.00000	0.67135	1.33338	Infinite	0.04732	0.015383
432	1.38386	8.15529	1.07013	5.8930	0.47730	0.037254
445	0.00000	2.59648	0.00000	Infinite	0.18302	0.026423
455	0.95951	3.98406	0.34305	4.1520	0.21319	0.089371

**Table 6:** Functional enrichment of biological processes in the human BPIFA1 protein network.

GO-term	Description	Count in Gene Set	False Discovery Rate
GO:0043030	regulation of macrophage activation	3 of 40	0.00086
GO:0051707	response to other organisms	6 of 1173	0.0045
GO:0034375	high-density lipoprotein particle remodeling	2 of 17	0.0052
GO:0043032	positive regulation of macrophage activation	2 of 23	0.0057
GO:0019730	antimicrobial humoral response	3 of 143	0.0057
GO:0010874	regulation of cholesterol efflux	2 of 21	0.0057
GO:0006955	immune response	6 of 1560	0.0057
GO:0009617	response to bacterium	4 of 555	0.0076
GO:0042742	defense response to bacterium	3 of 250	0.0098
GO:0042116	macrophage activation	2 of 41	0.0098
GO:0032720	negative regulation of tumor necrosis factor production	2 of 48	0.0098
GO:0019731	antibacterial humoral response	2 of 47	0.0098
GO:0006952	defense response	5 of 1234	0.0098
GO:0006869	lipid transport	3 of 272	0.0098
GO:0001818	negative regulation of cytokine production	3 of 245	0.0098
GO:0032496	response to lipopolysaccharide	3 of 298	0.0100
GO:0032677	regulation of interleukin-8 production	2 of 67	0.0116
GO:0015914	phospholipid transport	2 of 73	0.0127
GO:0098542	defense response to other organisms	4 of 859	0.0148
GO:0051704	multi-organism process	6 of 2514	0.0175
GO:0050829	antimicrobial humoral immune response	2 of 95	0.0188
GO:0061844	antimicrobial peptide	2 of 107	0.021
GO:0032675	regulation of interleukin-6 production	2 of 112	0.0234
GO:0071222	cellular response to lipopolysaccharide	2 of 146	0.0341
GO:0002274	myeloid leukocyte activation	3 of 574	0.0386
GO:0002699	positive regulation of the immune effector process	2 of 186	0.0492

**Table 7:** Recognition of protein-ligand binding sites of BPIFA1 using complementary comparisons of binding-specific substructures and sequence profile alignment.

	**COACH Results**			
	**C-Score**	**Cluster Size**	**Ligand Name**	**Predicted Binding Residue**
**BPIFA1**	0.04	3	PC1	51, 54, 64, 118, 120, 131, 133, 157, 163, 181, 219, 223, 227, 230, 231
	0.04	3	BPH	10, 14, 18
	0.04	3	1BP1A00	51, 54, 55, 57, 58, 168, 173, 174, 226, 227, 228, 231, 234, 237, 238
	0.03	2	XE	165, 235, 238, 239
	0.03	2	36E	200, 204
	0.03	2	DCW	187, 216, 219
	0.03	2	2CV	191, 204, 208, 211
	0.03	2	XE	5, 8, 9, 56, 57
	0.03	2	CRT	211, 215, 218
	0.03	2	3E8TA00	54, 58, 64, 84, 134, 137, 150, 155, 159, 188, 203, 211, 215, 227, 231
	**TM-site**			
	**C-score**	**Cluster size**	**Ligand name**	**Predicted binding residues**
**BPIFA1**	0.19	3	BPH(1),2CV(1)	10, 14, 18
	0.17	2	DCW(1)	187, 216, 219
	0.14	1	CRT(1)	211, 215, 218
	0.13	1	CLA	8, 12
	0.13	1	2CV	191, 204, 208, 211
	**S-site**			
	**C-score**	**Cluster size**	**Ligands name**	**Predicted binding residues**
**BPIFA1**	0.15	3	NEH, CLA, CU	216, 222, 223, 224, 225
	0.12	1	MG	179, 180
	0.10	1	FOL	215, 218

## Data Availability

All data relevant to this article shall be openly available to readers.

## Supplementary Materials

The following supporting information can be downloaded at: https://www.mdpi.com/article/10.3390/genes14010015/s1, Table S1: The species names and accession numbers used to study the BPIFA1 gene.
