# Supplementary material for: Molecular Evolution of the Bactericidal/Permeability-Increasing Protein (BPIFA1) Regulating the Innate Immune Responses in Mammals
Source: Genes (Basel). 2022 Dec 21;14(1):15. doi: 10.3390/genes14010015 (PMC9858190; doi:10.3390/genes14010015)
Supplement: Supplementary file 1 [file genes-14-00015-s001.zip › genes-2015138-supplementary.pdf]

Table S1. The species names and accession numbers used to study the BPIFA1 gene.

Sr. No.	Acession Number	Species Name
1	NM_033197.3	*Homo sapiens*
2	XM_007934725.1	*Orycteropus afer afer*
3	XM_042694769.1	*Dipodomys spectabilis*
4	XM_008703205.2	*Ursus maritimus*
5	XM_039863684.1	*Pteropus giganteus*
6	XM_037521140.1	*Talpa occidentalis*
7	XM_037143282.1	*Artibeus jamaicensis*
8	NM_011126.3	*Mus musculus*
9	XM_036321176.1	*Myotis myotis*
10	NM_172031.2	*Rattus norvegicus*
11	XM_025469732.1	*Canis lupus dingo*
12	NM_001301405.2	*Ovis aries*
13	XM_015457930.2	*Macaca fascicularis*
14	XM_045162581.1	*Bubalus bubalis*
15	XM_023251327.2	*Felis catus*
16	XM_007073573.2	*Panthera tigris*
17	XM_028827498.1	*Macaca mulatta*
18	XM_042930127.1	*Panthera leo*
19	NM_001005727.1	*Sus scrofa*
20	XM_041733220.1	*Vulpes lagopus*
21	XM_008703205.2	*Ursus maritimus*
22	NM_174426.3	*Bos taurus*
23	XM_003411504.2	*Loxodonta africana*
24	XM_038433510.1	*Canis lupus familiaris*
25	XM_036321176.1	*Myotis myotis*
26	XM_020151314.1	*Castor canadensis*
27	NM_001282365.1	*Chinchilla lanigera*
28	XM_007522683.1	*Erinaceus europaeus*
29	XM_005896919.2	*Bos mutus*
30	XM_013010681.1	*Dipodomys ordii*
31	XM_004668090.1	*Jaculus jaculus*
32	XM_004612568.1	*Sorex araneus*
33	XM_007446893.1	*Lipotes vexillifer*
34	XM_006881695.1	*Elephantulus edwardii*
